# Case report: Uncontrollable coronary artery perforation caused by severe stenosis proximal to loopy vessels

**DOI:** 10.3389/fcvm.2025.1609557

**Published:** 2025-07-01

**Authors:** Yuan Zeng, Lin Lu, Gang Wei, Wenwu Zheng, Gong Chen

**Affiliations:** ^1^ Department of Cardiology, The Affiliated Hospital of Southwest Medical University, Lu Zhou, Sichuan, China; ^2^ Institute of Cardiovascular Research, The Southwest Medical University, Luzhou, Sichuan, China

**Keywords:** coronary artery perforation, percutaneous coronary intervention, coronary artery bypass grafting, loopy vessels, covered stent

## Abstract

Coronary artery perforation(CAP) is a rare but potentially life-threatening event. Typically, such complications can be effectively managed with appropriately placed covered stents or embolization. However, in special cases, these methods may prove less effective. Here, we present a case of CAP requiring emergency surgical suturing: a perforation caused by radial cutting of the guidewire in a tortuous coronary lesion, accompanied by rapid opening of distal side branches in a narrowed vessel.

## Introduction

CAP is a life-threatening complication during interventional procedures, which can rapidly lead to cardiac tamponade, shock, or even death (1). Coronary arteries are generally straight and fixed in their course, but tortuous lesions—even those with over 360° loop-like configurations—do exist. When a stenosis is located at the extremely tortuous distal segment, the procedure becomes both technically challenging and high-risk. Most coronary perforations are caused by inappropriate balloon dilation or guidewire-induced injury to small vessels. Immediate sealing of the injury—including with covered stents or embolic agents—can help control the resulting pericardial effusion (2). Although pericardial effusion can be controlled, sealing the perforation may lead to occlusion of distal or branch vessels, resulting in myocardial necrosis in the corresponding area. In severe cases, this can cause large myocardial infarctions and even hemodynamic instability. Therefore, recognizing tortuous lesions with high perforation risk and selecting appropriate interventional strategies can help reduce the incidence of coronary perforation. This article presents the management of a perforation case as a reference for handling loop-like tortuous coronary lesions.

## Case presentation

**Figure 1:**
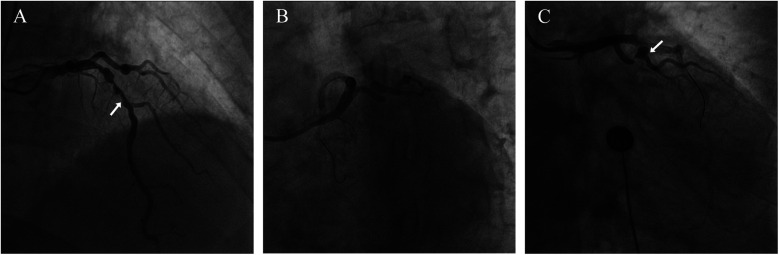
Coronary angiography results. **(A)** The arrow points to the LAD **(B)** severely stenosed and tortuous coronary artery **(C)** the arrow points to the loop-like vascular structure.

**Figure 2:**
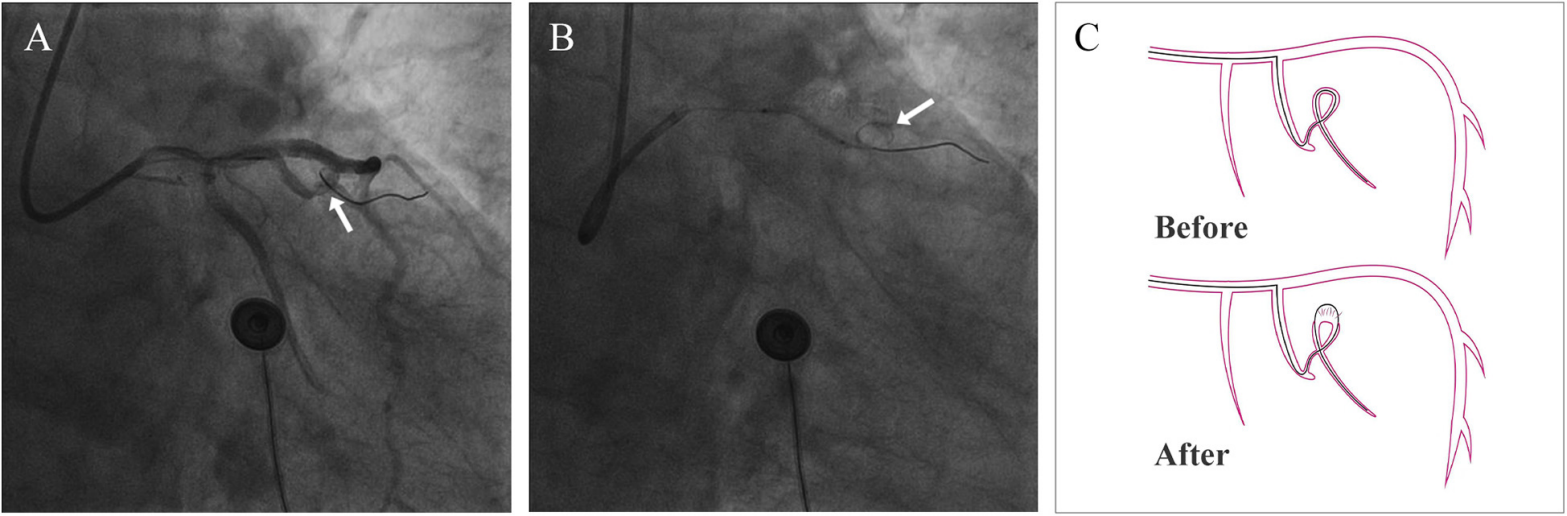
Coronary artery perforation. **(A)** The arrow points to the coiled guidewire before coronary artery rupture **(B)** the arrow points to the coiled guidewire after coronary artery rupture **(C)** schematic diagram of guidewire cutting the coronary artery.

**Figure 3:**
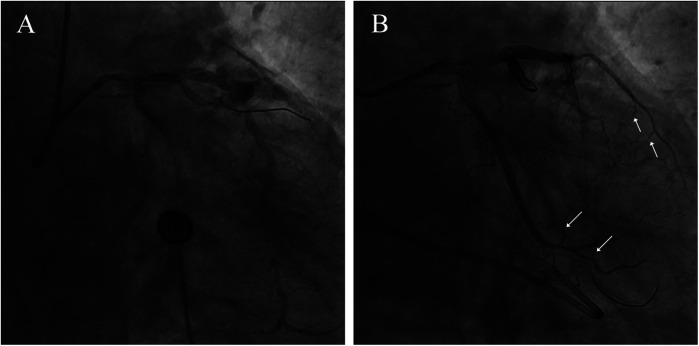
Confirmation of coronary artery rupture and interventional treatment. **(A)** Significant contrast extravasation **(B)** coil embolization of the ruptured vessel. The arrow points to the collaterals.

**Figure 4:**
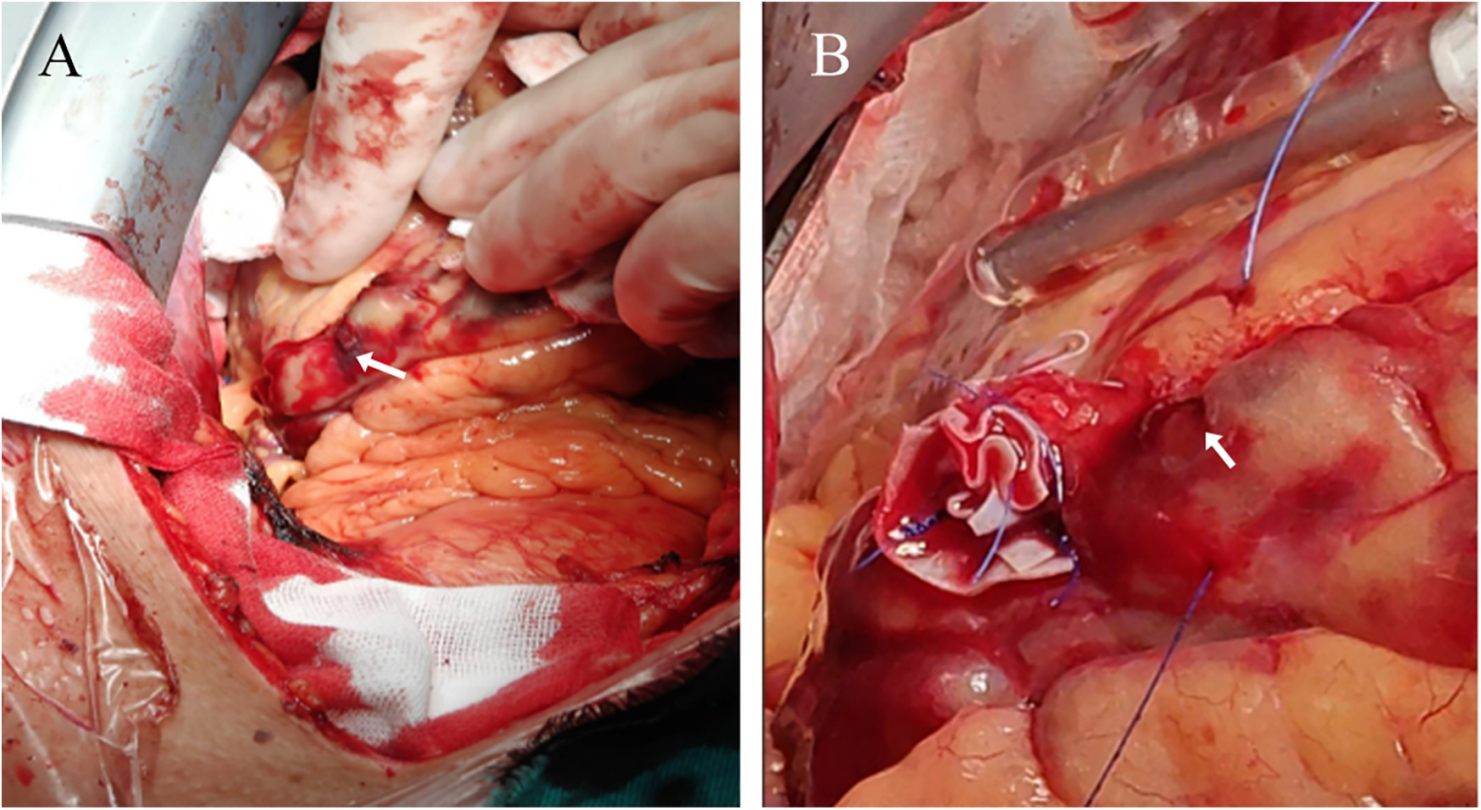
Surgical thoracotomy. **(A)** The arrow points to the coronary artery rupture site **(B)** the arrow points to the coronary artery rupture site that has not bled again after suturing.

A 74-year-old male patient was admitted due to dyspnea and chest pain for over one month. His medical history included hypertension and a 20-year smoking history. On admission, physical examination showed stable vital signs: body temperature 36.4°C, pulse rate 86 bpm, respiratory rate 20 breaths/min, and blood pressure 129/86 mmHg. There were no signs of heart failure or respiratory distress. Considering the patient's typical chest pain, T-wave inversion on the electrocardiogram, and elevated high-sensitivity troponin levels, a diagnosis of non–ST elevation myocardial infarction (NSTEMI) was established. Coronary angiography (CAG) revealed occlusion of the left circumflex artery (LCX), severe stenosis of the left anterior descending artery (LAD), and a highly tortuous high diagonal branch (forming a 120° angle with the main vessel), resulting in a distinct loop-like vascular structure (The imaging results can be found in the supplementary materials). The stenosis was located proximal to the looped segment, and multiple small coronary–ventricular fistulas were observed in the distal portion of the coronary artery (Figures 1A–C). The SYNTAX score of the affected vessels was over 33. We initially recommended coronary artery bypass grafting (CABG), but the patient declined. Subsequently, we proceeded with percutaneous coronary intervention (PCI). We first treated the LCX and LAD lesions, then addressed the diagonal branch. With microcatheter support, the guidewire was advanced through the severely narrowed segment to the distal vessel, where it was seen to coil helically in the distal stenotic area (Figure 2A). A 1.5 × 15 mm balloon was inflated at 18 atm for 4 s to dilate the lesion, followed by a 2.0 × 20 mm balloon, also inflated at 18 atm for 4 s. Upon deflation and withdrawal of the balloon, the previously coiled guidewire in the distal segment was seen to expand in diameter and shift forward (Figures 2B,C). Angiography revealed significant contrast extravasation, followed by the development of pericardial effusion. We believe that an Ellis type III CAP occurred at the distal portion of the diagonal branch. Due to difficulty in delivering a covered stent to the site of perforation, we attempted proximal sealing. Despite deploying coils to seal the ruptured proximal segment, rapid accumulation of pericardial effusion persisted. Angiography subsequently revealed the formation of multiple small distal side-branch collaterals originating from the ruptured vessel (Figures 3A,B), blood diverted from adjacent coronary branches to areas originally supplied by the diagonal branch. An emergency pericardiocentesis was performed by the surgical team, and 2,000 ml of blood was drained from the pericardial cavity. Due to the large perforation and the presence of collateral circulation, the proximal seal is ineffective, emergency surgical thoracotomy was deemed both feasible and necessary. Intraoperative exploration revealed a 6 mm rupture at the distal segment of the diagonal branch (Figure 4A), which was repaired using a pericardial patch with suturing, successfully stopping the bleeding (Figure 4B). Postoperatively, the patient received two units of whole blood to treat postoperative anemia, empirical antibiotics (piperacillin/tazobactam 4.5 g IV three times daily), and prophylactic anticoagulation with enoxaparin sodium 4,000 IU twice daily. The patient was discharged uneventfully two weeks after surgery. This case differs from typical balloon-induced vascular ruptures and is considered to be caused by guidewire-induced CAP with multiple collateral circulations. For this type of rupture, interventional therapy is often less effective, and surgical suturing offers a more definitive treatment option.

## Discussion

CAP is a rare but potentially fatal complication during PCI, with an incidence reported at 0.2%–0.5% (3, 4). The risk of CAP increases significantly in anatomically complex lesions, particularly in the presence of severe calcification, vascular tortuosity, or chronic total occlusion (CTO) (5). Ellis et al. classified CAP into three types based on angiographic appearance of the perforation (I, extraluminal crater without extravasation; II, pericardial or myocardial blushing; III, perforation > or = 1 mm diameter with contrast streaming; and cavity spilling). Type III represents the most severe form and carries the highest mortality rate, with cardiac tamponade reported in up to 40% of cases (6). The most common mechanism of CAP during PCI involves the guidewire entering small branches and causing vessel injury due to forceful advancement or rotation. Treatment options include a combination of hemostatic techniques such as coil embolization, covered stents, balloon tamponade, autologous tissue patches, and gelatin sponge (7, 8). Covered stents—especially those coated with polytetrafluoroethylene (PTFE)—are typically the first-line option, as they provide a durable seal to prevent blood leakage (9). However, in this case, the CAP was a Type III perforation caused by mid-shaft guidewire cutting, along with rapid formation of distal collateral circulation, posing a significant challenge for interventional hemostasis.

The distal portion of the guidewire beyond the first 30 cm is cylindrical and stiffer. It primarily serves as a delivery medium for balloons or stents and typically does not exert significant cutting force on the vessel wall. In this case, the guidewire followed the outer curvature of a looped coronary artery under high tension. As the balloon advanced toward the looped segment, the forward force transmitted through the balloon to the curved guidewire increased the mechanical stress on the vessel wall. When this external stress exceeded the vessel's mechanical tolerance, an Ellis type III perforation occurred.

In individuals suffering from stable ischemic heart disease, current guidelines strongly endorse the use of CABG over PCI, receiving a class I recommendation status (10). This clinical decision also applies to elderly patients over the age of 80 (11).

A meta-analysis comparing CABG and PCI in patients with multivessel coronary artery disease found that those with intermediate to high SYNTAX scores had significantly higher all-cause mortality 10 years after undergoing PCI (12). According to the 2020 ESC Guidelines for the management of acute coronary syndromes in patients without persistent ST-segment elevation, complete revascularization during the index PCI may be considered for NSTEMI patients with multivessel coronary artery disease (13). Therefore, based on this patient's CAG findings and weighing the survival benefit of CABG against the perioperative risks, CABG was recommended. Unfortunately, the patient refused surgery, and PCI was pursued instead. After treating the LCX and LAD lesions, we proceeded to address the diagonal branch lesion due to the presence of over 90% stenosis. In this case, the perforation occurred at the junction of a severely stenotic and tortuous coronary segment, making it difficult for the microcatheter to access the rupture site. As a result, conventional treatments such as balloon tamponade or covered stent deployment were not feasible. To achieve effective hemostasis, we attempted proximal sealing. Considering that covered stent placement may cause delayed endothelialization and restenosis, we opted to use coils for the occlusion (14). Unfortunately, the occlusion was ineffective. Emergency surgical intervention was ultimately required to achieve direct proximal vessel closure. To the best of our knowledge, this is the first reported case of CAP caused by radial wire cutting. This case underscores the indispensable role of surgery in managing complex and uncontrollable CAP. While most interventional teams are proficient in handling Type I or limited Type II CAPs, Type III perforations—particularly those located in anatomically challenging regions, associated with massive or uncontrollable bleeding, or inaccessible by guidewires or interventional devices—often exceed the limits of percutaneous approaches. Relying solely on PCI in such cases may delay definitive treatment. A multidisciplinary strategy, incorporating prompt cardiothoracic surgical support, is therefore essential. Furthermore, CAP frequently results in rapid pericardial blood accumulation or cardiac tamponade, which may necessitate urgent thoracotomy or surgical decompression. For lesions characterized by severe stenosis, looping, and tortuosity—especially when procedural risks are high—preprocedural risk assessment for perforation is critical. In such scenarios, CABG should be considered as an alternative when interventional strategies are likely to fail or become unsafe.

## Conclusion

There is a risk of CAP and rapid opening of collateral circulation during intervention for severe stenosis in looped (circumferential) coronary arteries, which can make hemostasis challenging. Therefore, extreme caution should be exercised when managing highly tortuous coronary lesions.

## Data Availability

The original contributions presented in the study are included in the article/Supplementary Material, further inquiries can be directed to the corresponding authors.

## References

[1] Hendry C Fraser D Eichhofer J Mamas MA Fath-Ordoubadi F El-Omar M Coronary perforation in the drug-eluting stent era: incidence, risk factors, management and outcome: the UK experience. EuroIntervention. (2012) 8(1):79–86. 10.4244/eijv8i1a13 22580251

[2] Abdalwahab A Farag M Brilakis ES Galassi AR Egred M. Management of coronary artery perforation. Cardiovasc Revasc Med. (2021) 26:55–60. 10.1016/j.carrev.2020.11.013 33203580

[3] Shimony A Joseph L Mottillo S Eisenberg MJ. Coronary artery perforation during percutaneous coronary intervention: a systematic review and meta-analysis. Can J Cardiol. (2011) 27(6):843–50. 10.1016/j.cjca.2011.04.014 21862280

[4] Kostantinis S Simsek B Karacsonyi J Alaswad K Krestyaninov O Khelimskii D Incidence, mechanisms, treatment, and outcomes of coronary artery perforation during chronic total occlusion percutaneous coronary intervention. Am J Cardiol. (2022) 182:17–24. 10.1016/j.amjcard.2022.07.004 36028387

[5] Teis A Fernández-Nofrerías E Rodríguez-Leor O Tizón H Salvatella N Valle V Coronary artery perforation by intracoronary guide wires: risk factors and clinical outcomes. Rev Esp Cardiol. (2010) 63(6):730–4. 10.1016/s1885-5857(10)70148-1 20515631

[6] Azzalini L Poletti E Ayoub M Ojeda S Zivelonghi C La Manna A Coronary artery perforation during chronic total occlusion percutaneous coronary intervention: epidemiology, mechanisms, management, and outcomes. EuroIntervention. (2019) 15(9):e804–e11. 10.4244/eij-d-19-00282 31217142

[7] Xenogiannis I Brilakis ES. Advances in the treatment of coronary perforations. Catheter Cardiovasc Interv. (2019) 93(5):921–2. 10.1002/ccd.28205 30953411

[8] Giannini F Candilio L Mitomo S Ruparelia N Chieffo A Baldetti L A practical approach to the management of complications during percutaneous coronary intervention. JACC Cardiovasc Interv. (2018) 11(18):1797–810. 10.1016/j.jcin.2018.05.052 30236352

[9] Lemmert ME van Bommel RJ Diletti R Wilschut JM de Jaegere PP Zijlstra F Clinical characteristics and management of coronary artery perforations: a single-center 11-year experience and practical overview. J Am Heart Assoc. (2017) 6(9):e007049. 10.1161/jaha.117.007049 28939719 PMC5634316

[10] Lawton JS Tamis-Holland JE Bangalore S Bates ER Beckie TM Bischoff JM 2021 Acc/Aha/Scai guideline for coronary artery revascularization: executive summary: a report of the American College of Cardiology/American Heart Association joint committee on clinical practice guidelines. Circulation. (2022) 145(3):e4–e17. 10.1161/cir.0000000000001039 34882436

[11] Kirov H Caldonazo T Riedel LL Tasoudis P Moschovas A Diab M Comparing outcomes between coronary artery bypass grafting and percutaneous coronary intervention in octogenarians with left main or multivessel disease. Sci Rep. (2023) 13(1):22323. 10.1038/s41598-023-49069-2 38102297 PMC10724226

[12] Chew NWS Koh JH Ng CH Tan DJH Yong JN Lin C Coronary artery bypass grafting versus percutaneous coronary intervention for multivessel coronary artery disease: a one-stage meta-analysis. Front Cardiovasc Med. (2022) 9:822228. 10.3389/fcvm.2022.822228 35402572 PMC8990308

[13] Collet JP Thiele H Barbato E Barthélémy O Bauersachs J Bhatt DL 2020 Esc guidelines for the management of acute coronary syndromes in patients presenting without persistent st-segment elevation. Eur Heart J. (2021) 42(14):1289–367. 10.1093/eurheartj/ehaa575 32860058

[14] Jana S. Endothelialization of cardiovascular devices. Acta Biomater. (2019) 99:53–71. 10.1016/j.actbio.2019.08.042 31454565

